# Evaluation of Software‐Optimized Protocols for Acoustic Noise Reduction During Brain MRI at 7 Tesla

**DOI:** 10.1002/jmri.29749

**Published:** 2025-03-06

**Authors:** Anton Glans, Linda Wennberg, Jonna Wilén, Lenita Lindgren, Pia C. Sundgren, Johan Mårtensson, Karin Markenroth Bloch, Boel Hansson

**Affiliations:** ^1^ Department of Nursing Umeå University Umeå Sweden; ^2^ Department of Diagnostics and Intervention, Radiation Physics Umeå University Umeå Sweden; ^3^ Department of Clinical Sciences Lund, Diagnostic Radiology, Faculty of Medicine Lund University Lund Sweden; ^4^ Department of Medical Imaging and Physiology Skåne University Hospital Lund Sweden; ^5^ Lund BioImaging Centre, Faculty of Medicine Lund University Lund Sweden; ^6^ Department of Clinical Sciences Lund, Logopedics, Phoniatrics and Audiology, Faculty of Medicine Lund University Lund Sweden

**Keywords:** hearing protection, MR safety, software optimization, ultra‐high field MRI

## Abstract

**Background:**

MR‐generated acoustic noise may be particularly concerning at 7‐Tesla (T) systems. Noise levels can be reduced by altering gradient output using software optimization. However, such alterations might influence image quality or prolong scan times, and these optimizations have not been well characterized.

**Purpose:**

To evaluate image quality, sound pressure levels (SPLs), and perceived noise levels when using the acoustic noise reduction technique SofTone for T_2_‐weighted fast spin echo (T_2_W FSE) and three‐dimensional T_1_‐weighted turbo field echo (3D T_1_W TFE), and to compare with conventional imaging during 7‐T brain MRI.

**Study Type:**

Prospective.

**Subjects:**

Twenty‐eight volunteers underwent brain MRI, with *n* = 26 for image quality evaluations.

**Field Strength/Sequence:**

Conventional and SofTone versions of T_2_W FSE and 3D T_1_W TFE at 7 T.

**Assessment:**

Peak SPLs (A‐weighted decibels, dBA), participant‐perceived noise levels (Borg CR10‐scale), qualitative image assessments by three neuroradiologists (four‐graded ordinal scales), interrater reliability, and percentage agreement.

**Statistical Test:**

Paired *t*‐test, Wilcoxon's Signed‐Rank Test, and Krippendorff's alpha; *p* < 0.05 were considered statistically significant.

**Results:**

SofTone significantly reduced peak SPLs: from 116.3 to 97.0 dBA on T_2_W FSE, and from 123.7 to 101.5 dBA on 3D T_1_W TFE. SofTone was perceived as significantly quieter than conventional scanning. T_2_W FSE showed no significant differences in image quality assessments (*p* = 0.21–1.00), except one radiologist noting significantly less artifact interference with SofTone. General image quality remained acceptable for 3D T_1_W TFE, though one radiologist scored it significantly lower with SofTone (mean scores: 3.08 vs. 3.65), and two radiologists observed significantly worse white and gray matter differentiation with SofTone (mean scores: 3.19 vs. 3.54; 2.27 vs. 2.81).

**Data Conclusion:**

SofTone can significantly reduce sound intensity and perceived noise levels while maintaining acceptable image quality with T_2_W FSE and 3D T_1_W TFE in brain MRI. It appears to be an effective tool for providing a safer, quieter 7‐T scan environment.

**Evidence Level:** 4

**Technical Efficacy:** Stage 5

**Abbreviations**
ANR: acoustic noise reduction
*B*_0_
: the static magnetic field
CNR: contrast‐to‐noise ratio
CSF: cerebrospinal fluid
dB/dBA: decibel/A‐weighted decibel
DTI: diffusion tensor imaging
FSE: fast spin echo (synonymous with turbo spin echo)
MPRAGE: magnetization‐prepared rapid gradient echo
PNS: peripheral nerve stimulation
SENSE: SENSitivity Encoding
SNR: signal‐to‐noise ratio
SPL: sound pressure level
T: Tesla
T_1_W/T_2_W: T_1_−/T_2_‐weighting
TE: echo time
TFE: turbo field echo
TR: repetition time
TSE: turbo spin echo (synonymous with fast spin echo)
ZTE: zero echo time

1

**Plain Language Summary**

- This study evaluates the viability of SofTone, an acoustic noise reduction technique, in reducing the loud noises generated during 7‐Tesla (T) brain MRI.
- We compared SofTone with conventional imaging for two different image weightings in 28 healthy volunteers.
- Our findings show that SofTone significantly reduces both sound pressure levels and participant‐perceived noise.
- For one image weighting, there was no difference in the overall image quality.
- For the other, minor image quality variations were observed using SofTone; however, the images remained diagnostically acceptable.
- SofTone appears to be an effective tool for providing a safer and quieter 7‐T scan environment.

## 2 Introduction

During an MRI exam, both acoustic noise and potential tissue stimulation are unwanted, yet inherent, side effects of utilizing the gradient coils [1]. During scanning, the interaction between the alternating gradient coil currents and the static magnetic field (*B*_0_) generates vibrational forces on the gradient‐coil conductors, causing loud hammering sounds. Routine scanning typically generates sound pressure levels (SPLs) that exceed the threshold for possible hearing impairment, 85 dB (dB), and some pulse sequences even reach 110–130 dB [1, 2, 3, 4]. Hearing protection is therefore required to minimize the risk of hearing damage [1, 2].

MR‐generated acoustic noise contributes to patient discomfort and anxiety [5, 6], decreased exam success rates [7, 8], and impedes patient‐radiographer communication [9, 10]. It has also been associated with stress and health‐related concerns among MR personnel [10, 11]. In addition, the swiftly alternating gradient fields during scanning induce currents that may stimulate conductive tissues in the exposed person, leading to peripheral nerve stimulation (PNS). PNS can manifest as uncontrolled muscle contractions, twitching, and tingling sensations in the limbs or torso, causing discomfort or even pain [1, 12, 13].

Decreasing the gradient activity can reduce both the acoustic noise and the gradient field exposure that induces PNS [14]. In practice, this reduction can be achieved through software‐optimized gradient‐restricting pulse sequences, which are typically provided by MR vendors as acoustic noise reduction (ANR) sequences—for example, “Whisper Mode” or “SofTone” [15, 16]. These techniques aim to smooth steep gradient current alterations, for example, by increasing the gradient ramp time or reducing its maximum amplitude (i.e., reducing the gradient slew rate), thereby decreasing the mechanical output of the gradient coil. However, these changes can restrict sequence parameters such as echo time (TE) and repetition time (TR), potentially reducing the image quality or extending the scan time [17, 18]. As ANR methods often require comprehensive user optimization [17, 18], they need to be validated and compared to conventional gradient output sequences before they are implemented for routine imaging [19].

In a previous study [14] we evaluated two ANR software solutions, which significantly decreased peak SPLs by up to 84% and gradient field exposure by 48%–66%, without reducing image quality. That study was performed using a 1.5‐Tesla (T) system. However, the demand for higher field MR systems is continuously increasing; a higher B_0_ provides an increased signal‐to‐noise ratio (SNR) and may enable improved image quality and diagnostic utility [20, 21]. These MR systems are typically utilized for high spatial or temporal resolution, which generally pushes the gradient system further and can cause higher vibration amplitudes and louder scans [22]. Therefore, hearing safety may be of particular concern with ultra‐high‐field MR systems, that is, ≥ 7‐T [4]. For instance, T_2_‐weighted turbo spin echo (TSE) and T_1_‐weighted magnetization‐prepared rapid gradient echo (MPRAGE) were recently studied in a 7‐T system, with reported peaks at 121.6 and 120.4 dB, respectively [4]. In another study, 63% of participants reported PNS to some degree during 7‐T MRI [23].

The aim of this study was to evaluate image quality, SPLs, and perceived noise levels when using the ANR technique SofTone for T_2_‐weighted fast spin echo (T_2_W FSE) and three‐dimensional T_1_‐weighted turbo field echo (3D T_1_W TFE), and to compare this to conventional imaging during 7‐T brain MRI.

## 3 Materials and Methods

This prospective single‐center study was approved by the Swedish Ethical Review Authority (DNR 2022‐05816‐01). Participation was voluntary, with written informed consent obtained, and could be withdrawn at any time. We employed a within‐subject design to compare software‐optimized ANR sequences for brain MRI with conventional counterparts. This study is part of a larger research project that aims to improve our understanding of software‐based ANR in ultra‐high‐field (7‐T) MRI, including prospective data on induced PNS, muscle activity, and other stress responses in the sympathetic nervous system. Evaluations of the non‐auditory aspects will be reported in future publications.

All scans took place using a 7‐T system (Achieva, Philips, Best, the Netherlands). Scanning was performed in first‐level controlled operating mode, with the specific absorption rate (SAR) limited to 4 W/kg for whole‐body or 3.2 W/kg for head. Each participant was examined head‐first in a supine position, using a 2 transmit (Tx)/32 receive (Rx) head coil (Nova Medical, Wilmington, MA, USA). To homogenize and increase the signal intensity in the cerebellum and mid‐brain area, we positioned dielectric pads between the head coil and the occipitotemporal region of each participant's head [24, 25].

Our evaluation focused on T_2_W FSE and 3D T_1_W TFE, as both are commonly used for morphological imaging in brain protocols at the study center. One conventional (non‐ANR) scan and one SofTone‐optimized scan were conducted for both. SofTone can be applied in five steps (factors), where each increment places additional restrictions on the gradients, achieving further noise reduction [15]. During optimization, we adjusted the parameters as little as possible to ensure that image contrast and scanning time remained similar to the conventional sequences. The SofTone sequences required a minor increase in the minimum allowed TE, for which we applied the shortest possible settings. Additionally, we increased the receiver bandwidth, resulting in a slight reduction in SNR. The echo spacing increased by 1.8 ms, from 12.0 to 13.8 ms, using SofTone on T_2_W FSE. Other parameters remained the same for the conventional and SofTone pairs (Table 1).

**TABLE 1:** Image parameters for the T_2_W FSE and 3D T_1_W TFE protocols.

Image parameter	Sequence			
	T_2_W FSE Conventional	T_2_W FSE SofTone	3D T_1_W TFE Conventional	3D T_1_W TFE SofTone
Slice thickness (mm)	1.0	1.0	0.7	0.7
Matrix (mm^2^)	440 × 366	440 × 366	304 × 343	304 × 343
Field of view (mm^2^)	220 × 190	220 × 190	240 × 210	240 × 210
Acquired voxel size (mm^3^)	0.50 × 0.52 × 1.0	0.50 × 0.52 × 1.0	0.70 × 0.71 × 0.70	0.70 × 0.71 × 0.70
Reconstructed voxel size (mm^3^)	0.29 × 0.29 × 1.0	0.29 × 0.29 × 1.0	0.36 × 0.36 × 0.35	0.36 × 0.36 × 0.35
Slice orientation	Transversal	Transversal	Sagittal	Sagittal
Slices	48	48	543	543
NSA	2	2	1	1
Acceleration factor (SENSE)	1.7	1.7	1.4 × 1.4	1.4 × 1.4
Turbo factor	9	9	250	250
Scan time (min:sec)	08:26	08:26	08:40	08:40
SofTone factor	—	3	—	4
Bandwidth (Hz/pxl)	125.9	142.6	252.5	336.6
TR/TE (ms)	5060/60	5060/69	8.0/2.7	8.0/3.8
Inversion time (ms)	—	—	1200	1200
Flip angle (°)	90	90	7	7
Echo spacing (ms)	12.0	13.8	—	—
Shot duration/interval (ms)	—	—	2210/3500	2210/3500

Abbreviations: 3D T_1_W TFE, three‐dimensional T_1_‐weighted turbo field echo; Hz/pxl, Hertz/pixel; NSA, numbers of signal acquisitions; SENSE, SENSitivity Encoding (used in both phase × slice directions in 3D imaging); T_2_W FSE, T_2_‐weighted fast spin echo; TE, echo time; TR, repetition time.

The protocol also included a survey scan (time; min:sec; 00:57), a coil reference scan (00:11), a SENSitivity Encoding (SENSE) reference scan (00:38), and a *B*_0_ shimming (00:18). In addition, the participants were examined using both conventional (05:42) and SofTone (05:42) diffusion tensor imaging (DTI); however, that data will be presented in a separate publication. The order of the T_2_W FSE, 3D T_1_W TFE, and DTI sequences was randomized for each participant, and participants were blinded to the specific sequences being used to mitigate bias.

Participants were healthy volunteers recruited through advertisements in the study center, hospital, and university area, and via local contacts in the researchers' networks. The exclusion criteria were non‐MRI‐compatible implants, being < 18 years old, pregnancy, and not meeting the clinic's MRI safety criteria. Additionally, participants were required to have functional hearing to be able to evaluate perceived noise levels and to enable verbal communication via the intercom system. Since the head coil was too narrow to fit headphones, the participants were required to use two layers of hearing protection: disposable earplugs and a sound‐attenuating impression paste applied to their auricles (see Figure 5.1 in [26]). All morphological MR images were interpreted by a radiologist to ensure no incidental pathologies were missed and, where necessary, reported for further follow‐up. Data were collected between March and April 2023.

### 3.1 Acoustic Noise Assessments

We used the same acoustic noise assessments as those previously conducted at 1.5‐T [14]. An MRI‐compatible sound level meter (OptiSLM 100; Optoacoustics Ltd., Mazor, Israel) was used to assess peak SPLs in A‐weighted decibels (dBA), with the microphone (OPTIMIC 1155) taped to the participant's jugular notch. The sound level meter was set at the high‐level range (65–130 dBA) and a max hold response rate (decay < 1 dB/3 min). We calculated the difference in mean peak SPLs, ∆L, by subtracting the conventional (*A*) and the SofTone (*B*) pair: ∆*L* = *A*−*B*. To linearly compare the mean SPLs between the same weighted pairs, the differences in sound pressure, ∆SP, were then converted from dB to Pascal (Pa), using $∆\mathrm{SP}=10^{∆L/20}$ [14, 27], subsequently enabling us to calculate the percentage differences between SofTone and conventional scanning.

The Borg CR10 scale was used to evaluate how the participants perceived noise levels during the pulse sequences. Borg CR10 is a categorical ratio scale that ranges from 0 to 10 (allowing decimals) and is validated for assessing perceptions and feelings [28]. For this study, 0 signified complete silence, and 10 the loudest sound the participant had ever experienced. Should the participants perceive the noise level to exceed 10, they could use > 11 as a new absolute maximum [28]. The scale was explained in the preparation area before the exam started and was visible on a monitor during the exam via the head coil mirror. Immediately following each sequence, we used the speaker system to ask the participants to verbally rate their experience of the noise level.

### 3.2 Image Quality Assessments

Image quality was evaluated qualitatively by three neuroradiologists, all well‐versed in 7‐T MRI; Reader 1 (P.C.S) and Reader 3 had both 26 years of experience in neuroradiology, and Reader 2 had 10 years of experience. They assessed images across four domains: the presence and extent of artifacts, general image quality, ability to differentiate between white and gray matter, and ability to differentiate cerebrospinal fluid (CSF) from surrounding soft tissues. Artifacts were assessed using an ordinal scale ranging from 1 (unreadable) to 4 (no artifacts). When an artifact that could potentially interfere with image interpretation was identified, the artifact type was specified on a separate item. The general image quality and tissue differentiations were graded from poor [1] to excellent [4]. The scale items, inspired by previous publications in similar contexts [14, 29], were discussed with the radiologists prior to finalizing the definitions and terminology (see Supporting Information). Assessments were performed individually by the readers, and images were randomized and blinded to mitigate bias. Assessment scores ≥ 2 were considered diagnostically acceptable, indicating that the images provided sufficient quality for diagnostic interpretation despite any limitations.

### 3.3 Statistical Analysis

All statistical testing was performed in SPSS version 27 (IBM Corp., Armonk, NY), using a *p*‐value ≤ 0.05 to indicate statistical significance.

We performed an a priori power calculation to determine the sample size, taking our larger research project scope into account (which also considered PNS assessments). Assuming a 63% prevalence of PNS during conventional scanning [23], G*Power (v. 3.1.9.4) suggested that 28 participants would achieve a power > 80% (alpha = 0.05, two‐tailed). Based on the mean difference in SPLs between ANR scanning and conventional scanning previously observed at 1.5 T [14], a sample of 28 participants yielded a power > 99% (alpha = 0.05, two‐tailed).

Peak SPLs and Borg CR10 scores were analyzed using a paired t‐test, provided the data adhered to a normal distribution. The normality of the distribution was tested using the Wilks‐Shapiro method due to the relatively small size of our sample [30]. In cases where the data was not normally distributed, a non‐parametric Wilcoxon's signed‐rank test was used instead. Qualitative assessments of image quality were also analyzed using Wilcoxon's signed‐rank test, where each assessment item between both sequence pairs was tested for each reader individually.

To test interrater reliability, we calculated Krippendorff's alpha (*α*) using 10,000 bootstrap samples to weigh the observed values and to account for chance [31]. The *α*‐value, which ranges between 0.000 (absence of reliability) and ± 1.000 (perfect reliability), indicates the agreement between the three readers. A negative *α*‐value indicated systematic disagreement. We also conducted pairwise comparisons between each reader using percent agreement.

## 4 Results

Our study included 28 healthy volunteers. We scanned an additional participant who could not complete the examination due to uncomfortable sensations at the site of a metallic orthopedic implant. For safety reasons, we stopped the examination and excluded this participant, and recruited another participant as a replacement. Among the final cohort of 28 participants, 20 identified as women and eight as men (age range 18–56; mean ± standard deviation, SD: 33 ± 11 years). The images of two participants exhibited motion artifacts throughout their examinations that noticeably degraded the image quality of at least one sequence among the same‐weighted conventional and SofTone pairs. To ensure a consistent basis for the evaluation of image quality, we excluded these two participants from all image‐quality assessments, resulting in *n* = 26 for those evaluations. However, we included these two participants in the SPL and Borg CR10 evaluations (*n* = 28).

### 4.1 Sound Pressure Level

**FIGURE 1:**
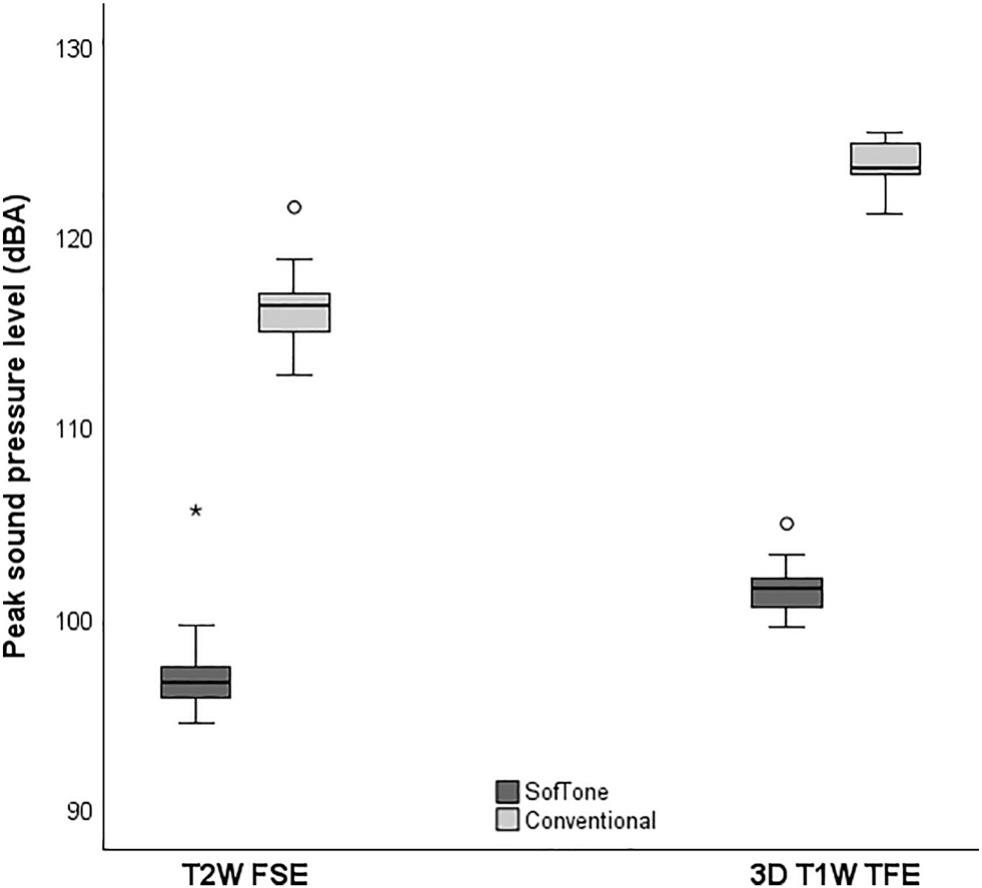
Box plot showing the distribution of peak sound pressure levels in A‐weighted decibels (dBA) for T_2_‐weighted fast spin echo (T_2_W FSE) and three‐dimensional T_1_‐weighted turbo field echo (3D T_1_W TFE), with and without SofTone (*n* = 28). The black centerlines show the median; the boxes show the data ranges between the first and third quartiles; dark gray boxes represent SofTone scanning; light gray boxes represent conventional scanning; * = extreme outlier; ° = mild outlier. While the cause of these outliers remains unclear, potential *pressure doubling* [17] cannot be ruled out, that is, reflected sound waves undergoing in‐phase enhancements in the vicinity of the scanned participant.

Significant differences were observed in the peak SPLs between SofTone and conventional scanning for both T_2_W FSE and 3D T_1_W TFE. The recorded peak SPL during T_2_W FSE conventional scanning was 116.3 ± 1.8 dBA (mean ± SD), and 97.0 ± 2.1 dBA during T_2_W FSE SofTone (significant). This represents a mean difference of 19.3 dBA, corresponding to an 89% reduction in sound pressure with the application of SofTone. For 3D T_1_W TFE, the mean peak SPL was 123.7 ± 1.2 dBA for conventional scanning and 101.5 ± 1.2 dBA for SofTone scanning (significant). This difference of 22.2 dBA translates to a 92% reduction in sound pressure. The SPL distributions are presented in Figure 1.

### 4.2 Perceived Noise Level

The participants perceived that both SofTone sequences were significantly quieter than the conventional scanning. On the Borg CR10 scale, the participants rated the conventional T_2_W FSE sequence as close to ‘strong/loud’ (4.5 ± 1.7) on average. In contrast, T_2_W FSE SofTone was perceived as close to ‘moderate’ (3.1 ± 1.1). For 3D T_1_W TFE, the conventional sequence was rated between ‘strong’ and ‘very strong’ (5.7 ± 2.0), and SofTone between ‘moderate’ and ‘strong’ (3.9 ± 1.7).

### 4.3 Image Quality

For T_2_W FSE, no significant differences were observed in general image quality, ability to differentiate between white matter and gray matter, and ability to differentiate CSF from adjacent soft tissues (*p* ranging from 0.21 to 1.00, see Table 2). Interestingly, Reader 2 rated SofTone as having significantly fewer interfering artifacts than the conventional sequence, although the mean score only differed by 0.16. Reader 1 and Reader 3 did not observe any significant differences in artifacts between SofTone and conventional scanning (Reader 1: *p* = 1.00; Reader 3: *p* = 0.74).

**TABLE 2:** Image‐quality assessments for T_2_W FSE (*n* = 26).

	Sequence		*p*
	T_2_W FSE Conventional	T_2_W FSE SofTone	
Reader 1			
Artifacts	3.12 ± 0.43	3.12 ± 0.52	1.00
General image quality	3.42 ± 0.58	3.35 ± 0.63	0.64
White matter from gray matter	3.50 ± 0.58	3.50 ± 0.65	0.98
CSF from soft tissues	3.96 ± 0.20	3.96 ± 0.20	1.00
Reader 2			
Artifacts	2.88 ± 0.43	3.04 ± 0.45	**< 0.05**
General image quality	2.92 ± 0.69	3.00 ± 0.49	0.59
White matter from gray matter	3.50 ± 0.58	3.35 ± 0.63	0.32
CSF from soft tissues	3.85 ± 0.46	3.88 ± 0.33	0.74
Reader 3			
Artifacts	2.77 ± 0.43	2.73 ± 0.45	0.74
General image quality	2.69 ± 0.55	2.58 ± 0.50	0.37
White matter from gray matter	2.69 ± 0.55	2.54 ± 0.51	0.21
CSF from soft tissues	2.92 ± 0.27	2.92 ± 0.27	1.00

*Note*: The distribution of image assessment scores (range: 1.00–4.00) shown with mean ± standard deviation. Bold values indicate statistical significance.

Abbreviations: CSF, cerebrospinal fluid; T_2_W FSE, T_2_‐weighted fast spin echo.

**FIGURE 2:**
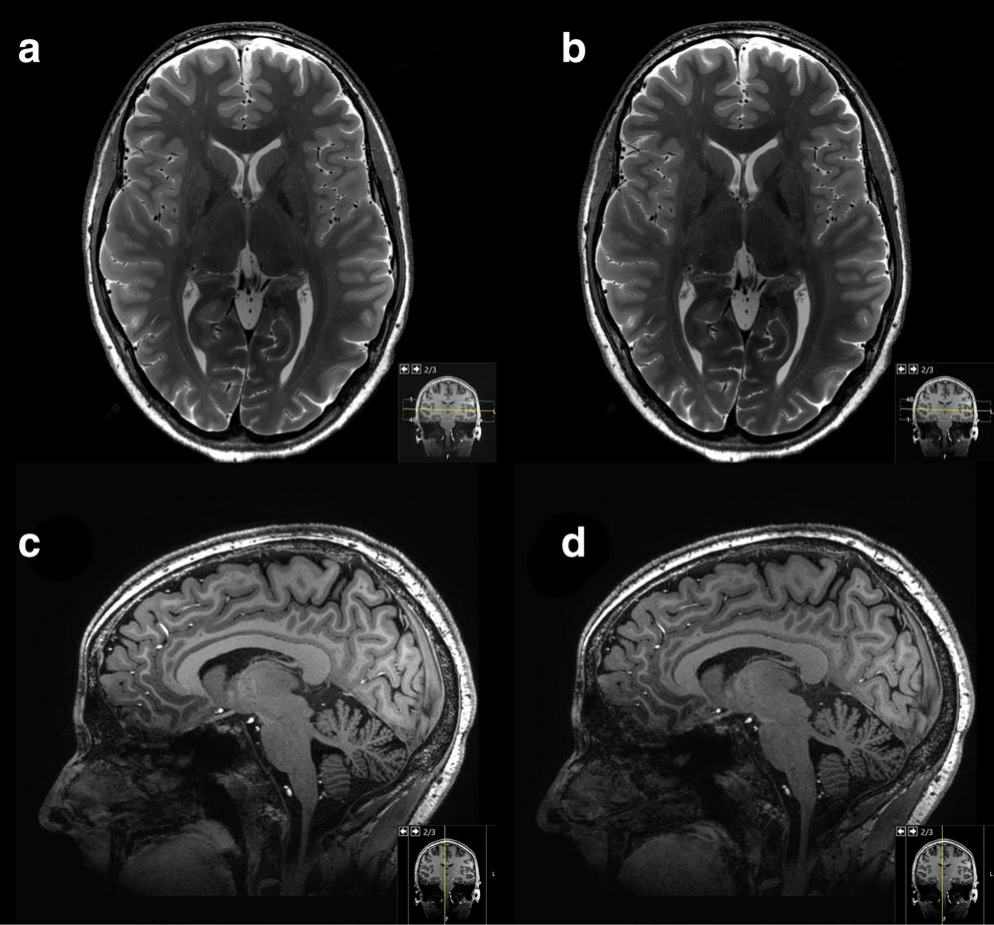
Example images produced using T_2_‐weighted fast spin echo (T_2_W FSE) and three‐dimensional T_1_‐weighted turbo field echo (3D T_1_W TFE) from a healthy volunteer. (a) T_2_W FSE Conventional, 116.1 dBA (peak sound pressure level in A‐weighted decibels); (b) T_2_W FSE SofTone, 97.4 dBA; (c) 3D T_1_W TFE Conventional, 123.5 dBA; (d) 3D T_1_W TFE SofTone, 101.2 dBA. The three radiologists rated the general image quality of all of these images as either “3, good” or “4, excellent”. Images reproduced with permission from Glans [32].

The image quality for 3D T_1_W TFE exhibited a slightly higher subjective variance among the three radiologists (Table 3). While no significant differences were observed in terms of artifacts or ability to differentiate CSF from soft tissues (*p* ranging from 0.05 to 0.32), Reader 1 rated the general image quality of SofTone (mean ± SD: 3.08 ± 0.63) as significantly inferior to that of conventional scanning (mean ± SD: 3.65 ± 0.56). Neither of the other two readers observed any significant differences in general image quality (Reader 2: *p* = 0.13; Reader 3: *p* = 0.08). However, Readers 2 and 3 found the differentiation between white and gray matter to be significantly worse in 3D T_1_W TFE SofTone (mean ± SD, Reader 2: 3.19 ± 0.63; Reader 3: 2.27 ± 0.45) than in conventional scanning (mean ± SD, Reader 2: 3.54 ± 0.58; Reader 3: 2.81 ± 0.40), whereas Reader 1 did not (*p* = 0.06). An example image is shown in Figure 2.

**TABLE 3:** Image‐quality assessments for 3D T_1_W TFE (*n* = 26).

	Sequence		*p*
	3D T_1_W TFE Conventional	3D T_1_W TFE SofTone	
Reader 1			
Artifacts	2.92 ± 0.39	2.77 ± 0.43	0.16
General image quality	3.65 ± 0.56	3.08 ± 0.63	**< 0.01**
White matter from gray matter	3.85 ± 0.37	3.62 ± 0.57	0.06
CSF from soft tissues	4.00 ± 0.00	3.96 ± 0.20	0.32
Reader 2			
Artifacts	3.00 ± 0.28	2.88 ± 0.43	0.18
General image quality	3.04 ± 0.34	2.85 ± 0.54	0.13
White matter from gray matter	3.54 ± 0.58	3.19 ± 0.63	**0.02**
CSF from soft tissues	3.77 ± 0.43	3.50 ± 0.51	0.05
Reader 3			
Artifacts	2.88 ± 0.33	2.69 ± 0.55	0.13
General image quality	2.73 ± 0.45	2.50 ± 0.51	0.08
White matter from gray matter	2.81 ± 0.40	2.27 ± 0.45	**< 0.001**
CSF from soft tissues	3.00 ± 0.00	2.88 ± 0.33	0.08

*Note*: The distribution of image assessment scores (range: 1.00–4.00) shown with mean ± standard deviation. Bold values indicate statistical significance.

Abbreviations: CSF, cerebrospinal fluid; 3D T_1_W TFE, three‐dimensional T_1_‐weighted turbo field echo.

The interrater agreement and reliability scores are shown in Table 4. Overall, the agreement between the three readers was low: no percent agreement exceeded 77%, with the highest *α* being −0.3753, corresponding to a fair systematic disagreement in assessment.

**TABLE 4:** Reliability and agreement between observers (*n* = 26).

Image assessment	Sequence			
	T_2_W FSE Conventional	T_2_W FSE SofTone	3D T_1_W TFE Conventional	3D T_1_W TFE SofTone
Artifacts	*α* = 0.3084	*α* = 0.2719	*α* = −0.0268	*α* = 0.2745
	76%	64%	77%	71%
	(20/26; 18/26; 21/26)	(16/26; 16/26; 18/26)	(20/26; 19/26; 21/26)	(17/26; 19/26; 19/26)
General image quality	*α* = 0.2604	*α* = 0.0453	*α* = 0.0027	*α* = 0.2093
	53%	46%	40%	49%
	(15/26; 8/26; 18/26)	(12/26; 8/26; 15/26)	(8/26; 4/26; 18/26)	(10/26; 10/26; 18/26)
White matter from gray matter	*α* = 0.1473	*α* = −0.1223	*α* = −0.1477	*α* = −0.1515
	40%	33%	32%	29%
	(19/26; 7/26; 5/26)	(10/26; 7/26; 8/26)	(15/26; 2/26; 8/26)	(16/26; 3/26; 3/26)
CSF from soft tissues	*α* = −0.2446	*α* = −0.3753	*α* = −0.3601	*α* = −0.3086
	33%	32%	33%	31%
	(23/26; 0/26; 2/26)	(22/26; 1/26; 2/26)	(20/26; 0/26; 6/26)	(12/26; 1/26; 11/26)

*Note*: The interrater agreement between qualitative assessments made by the three radiologists: Reader 1 (R1), Reader 2 (R2), and Reader 3 (R3). Data shown in top row, Krippendorff's alpha (*α*); in middle row, percent agreement (%); in bottom row, total number of agreements out of a maximum *n* = 26 between R1/R2; R1/R3; R2/R3.

Abbreviations: CSF, cerebrospinal fluid; 3D T_1_W TFE, three‐dimensional T_1_‐weighted turbo field echo; T_2_W FSE, T_2_‐weighted fast spin echo.

**FIGURE 3:**
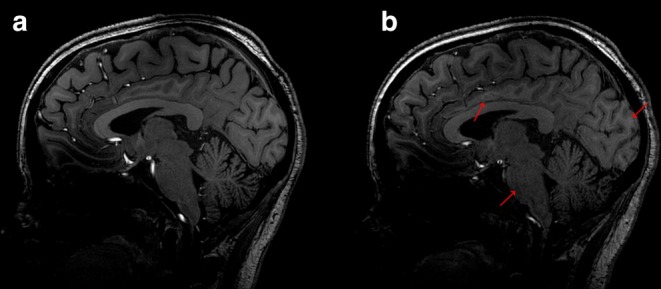
Example images from a healthy volunteer, illustrating image quality differences between conventional and SofTone scanning with three‐dimensional T_1_‐weighted turbo field echo (3D T_1_W TFE). (a) Conventional, 122.2 dBA (peak sound pressure level in A‐weighted decibels); (b) SofTone, 100.7 dBA. Red arrows illustrate slightly more image blurring, contours, and signal loss using SofTone compared to the conventional imaging, along with less detailed white and gray matter discrimination. For SofTone, the white and gray matter discrimination and the general image quality were rated as either “3, good” or “2, fair”, whereas the ratings for the conventional sequence were either “4, excellent” or “3, good”.

Artifacts were identified in all images; these were typically related to 7‐T‐specific characteristics such as *B*_1_ inhomogeneity, susceptibility artifacts (e.g., sinus nasale), or Gibbs' artifacts [25]. These artifacts, while noticeable, were not necessarily deemed to hinder the overall diagnostic utility of the morphological information. However, they did affect certain areas of the image, reducing the general image quality assessment scores given by all three readers, as well as the differentiation of CSF from soft tissues. Additionally, some assessments revealed the presence of motion, chemical shift, and/or flow artifacts. Figure 3 illustrates an example where increased signal loss, motion, and image blurring were noticed on 3D T_1_W TFE SofTone compared to the conventional scanning, along with a decreased ability to differentiate between white and gray matter.

## 5 Discussion

The use of SofTone in 7‐T brain MRI was found to significantly reduce both peak SPLs and perceived noise levels while maintaining acceptable subjective image quality.

No significant differences in image quality were observed between conventional and SofTone T_2_W FSE, although one of the radiologists noted slightly less artifact interference with SofTone. This minor improvement could possibly be attributed to the increased receiver bandwidth, potentially reducing chemical shift and susceptibility artifacts [33, 34]. Several studies investigating various ANR software and B_0_ field strengths have shown preserved image quality with T_2_W FSE/TSE [14, 27, 35, 36, 37, 38]. Some of these have evaluated algorithmic software that optimizes and smooths gradient forms, which could offer even quieter exams [14, 37]. Since not all MR systems have access to such software, it is useful to know that plain slew rate restriction is still effective in 7‐T MRI.

Despite greater variance in the assessed image quality for 3D T_1_W TFE, as evidenced by the two radiologists who found SofTone to provide worse white and gray matter differentiation, their ratings remained within an acceptable diagnostic range. However, the loss of contrast between white and gray matter may have been caused by the slightly longer TE, allowing more T_2_*‐relaxation and, thus, less T_1_‐contrast. Jacobs et al. [39] also found that the contrast‐to‐noise ratio (CNR) between gray and white matter decreased when using an ANR‐optimized T_1_W MPRAGE combined with a silent gradient coil. Similarly, software‐based ANR fluid‐attenuated inversion recovery (FLAIR) resulted in decreased CNR at 7 T [40]. The assessment of general image quality significantly differed by one radiologist in our study, where the mean score for 3D T_1_W TFE SofTone was closer to “good” compared to “excellent” for the conventional sequence. While the quality is still acceptable for diagnostics, the results suggest uncertainty regarding how software‐optimized ANR potentially influences subjective preferences.

Another effective ANR software approach for 3D T_1_W gradient echo sequences, including TFE and MPRAGE, is the “zero echo time” (ZTE) technique [41]. ZTE utilizes sub‐millisecond TEs and continuously applied gradients [42] to achieve near‐ambient noise levels, and reduced SPLs by 27–29 dB as compared to conventional T_1_W MPRAGE [43, 44]. However, ZTE may limit user customization, for example, requiring a cubic field of view or disabling acceleration factors [41], and it inherently struggles to provide high image quality with T_2_W or diffusion weighting [42]. Notably, ZTE is not an option on all systems, including the one used in this study, which complicates direct comparisons. Nonetheless, there is a clear contrast between our study and others in terms of the sound level of the conventional sequences: our T_1_W TFE peaked at 123.7 dBA, which was considerably louder than the 78 and 87 dB recorded for conventional T_1_W MPRAGE in previous ZTE studies at 3‐T [43, 44]. Despite SofTone reducing SPLs by up to 22.2 dBA in our study, the scanner's noise level still necessitates disposable hearing protection, as the system's head coil is too small for headphones [24]. However, since every 3 dB decrease corresponds to a halving of sound intensity [45], the SPL reduction achieved with SofTone here effectively extends permissible scan time nearly sevenfold, making it a very useful tool for hearing protection during lengthy 7‐T exams.

The average peak SPL reduction of 89%–92% aligns with our participants' scores for noise perception, rating SofTone as quieter than conventional scanning. This noise reduction not only decreases the risk of hearing damage, but may enhance patient comfort [35, 36, 46]. Additionally, patients have previously experienced less discomfort and an improved ability to hear music during MRI at 1.5‐T and 3‐T with ANR sequences [47]. In another study, patients reported the overall experience of undergoing 7‐T MRI as significantly less comfortable than healthy volunteers [23] and suggested improvements such as receiving information prior to particularly loud sequences and better hearing protection. Consequently, the Borg CR10 scores reported by the healthy volunteers in our study might underestimate the discomfort experienced in clinical contexts (i.e., by patients). ANR sequences could be especially helpful for vulnerable patient groups, such as children, claustrophobic and non‐cooperative patients, those under sedation, and patients with tinnitus or other auditory sensitivities [7, 33, 46, 48].

Technical details regarding SofTone are sparse in the literature. However, our practical experience indicates that some sequence parameters (e.g., TE, TR, echo spacing, and receiver bandwidth) increase when applying the software, although the extent of this depends on the SofTone factor. Our study used SofTone factors 3 and 4, suggesting that a lower factor could still significantly reduce the SPL—albeit slightly louder than our current results show—but with even less impact on image quality. An updated implementation of SofTone is available on the vendor's MR platform, named ComforTone, in which optimization of ANR settings is automated [15]. Still, there are challenges that need to be addressed [33].

The variety of ANR software available for commercial MR systems offers alternatives, but the potential compromises in image quality and prolonged scanning time for a specific sequence introduce uncertainty. Future technological advancements, including artificial intelligence (AI), can hopefully address these issues. Notably, Swedish MR radiographers desired more user‐friendly solutions to increase the clinical adoption of software‐based ANR [10]. Despite its promising utility, current ANR software may influence contrast‐determining parameter changes, and we recommend testing ANR on your local system before clinical implementation to ensure its viability. Given earplugs do not always fit and a second layer of hearing protection can only account for up to 5 dBA of extra dampening [45], reducing SPLs by 19–22 dBA through ANR software is a powerful way of minimizing the risk of hearing damage and increasing patient comfort.

### 5.1 Limitations

The potential changes in tissue contrast and signal intensity from utilizing SofTone could conceivably affect quantitative interpretations, such as voxel‐based morphometry [49]. Although such evaluations were not within the scope of this initial study, they present an interesting avenue for future research. We did not evaluate quantitative image quality, for example, SNR or CNR, since our parameter changes in receiver bandwidth and TE inherently resulted in a lower relative SNR for SofTone sequences. Furthermore, the use of parallel imaging (SENSE) made the background noise inhomogeneous and unreliable for fair assessments. Nevertheless, the primary focus was assessing whether software‐based ANR affected image quality as interpreted by radiologists. Future research could explore its effects on pathology visualization in patient scanning.

Given the lack of standardized evaluations of brain MRI, we developed our own items, taking inspiration from previous work [14, 29]. Our decision to assess the whole brain may have influenced the outcome, given that 7‐T imaging can cause notable signal losses in local areas [25]. While evaluating specific brain areas might have provided a more robust overview, such separation would not have fully covered the intended clinical use for morphological imaging. Therefore, we chose not to separate local areas of the brain for the overview assessments. Our analysis was extended by assessing contrast differentiation between white and gray matter and CSF from surrounding soft tissue.

Another limitation is the low interrater reliability. The skewed distributions of the radiologists' assessments, where some item values were infrequently used (e.g., a near‐absence of scores of “1”), may have contributed to a lower Krippendorff's *α* [50]. Individual bias may also have been a factor, in that different interpretations existed between the radiologists. This could possibly be attributed to individual preferences in terms of image presentation or ambiguous item definitions. Although we developed the items in dialogue with the three radiologists and provided them with the written item definitions during their assessments, ideally, we could have also provided training or calibration exercises to mitigate bias. Still, we argue that the pairwise sequence comparisons of each radiologist individually, inherent to the within‐subject design, constitute the critical outcome for this initial assessment.

Lastly, the fact that our participants were also examined with two DTI sequences could have influenced their noise perception of T_2_W FSE and 3D T_1_W TFE. To mitigate this, we randomized the order of the six sequences.

### 5.2 Conclusion

Using SofTone in 7‐T brain MRI can significantly reduce the peak SPLs and participant‐perceived noise levels while maintaining acceptable subjective image quality with T_2_W FSE and 3D T_1_W TFE. The reduction in sound intensity provides a safer and more comfortable scan environment and is an effective tool for hearing protection during potentially lengthy MR protocols.

## Supporting information


**Data S1.** Supporting Information.
