# Supplementary material for: Evaluation of Software‐Optimized Protocols for Acoustic Noise Reduction During Brain MRI at 7 Tesla
Source: J Magn Reson Imaging. 2025 Mar 6;62(2):577–87. doi: 10.1002/jmri.29749 (PMC12276638; doi:10.1002/jmri.29749)
Supplement: Supplementary file 1 — Data S1. Supporting Information. [file JMRI-62-577-s001.pdf]

Image assessment	Scoring system
1. Image artifacts	1, Unreadable; non-diagnostic examination due to artifacts.<br>2, Major artifacts interfering image interpretation.<br>3, Minor artifacts slightly interfering image interpretation.<br>4, No artifacts interfering image interpretation.
2. General image quality	1, Poor: poor image quality, impossible to diagnostically interpret images. Non-diagnostic; re-scanning needed.<br>2, Fair: suboptimal anatomical delineation but offers diagnostic anatomical definition; sufficient image quality with diagnostic limitations.<br>3, Good: well delineated anatomical structures with good internal anatomy preservation; standard examination without diagnostic limitations.<br>4, Excellent: very well delineated and contrasted anatomy between structures and excellent internal anatomy preservation; no diagnostic limitations.
3. Ability to differentiate between white and grey matter	1, Poor; indistinct anatomical delineation and disturbed internal structure; non-diagnostic.<br>2, Fair; suboptimal anatomical delineation but offers diagnostic anatomical definition; diagnostic limitations.<br>3, Good; well delineated anatomical structures between white and grey matter with good internal anatomy preservation; standard examination without diagnostic limitations.<br>4, Excellent; very well delineated and contrasted anatomy between white and grey matter, and excellent internal anatomy preservation; no diagnostic limitations.
4. Ability to differentiate CSF from surrounding soft tissues	1, Poor; indistinct anatomical delineation and disturbed internal structure; non-diagnostic.<br>2, Fair; suboptimal anatomical delineation but offers diagnostic anatomical definition; diagnostic limitations.<br>3, Good; well delineated anatomical structures between CSF and surrounding soft tissues with good internal anatomy preservation; standard examination without diagnostic limitations.<br>4, Excellent; very well delineated and contrasted anatomy between CSF and surrounding soft tissues, excellent internal anatomy preservation; no diagnostic limitations.
